# Editorial: Health systems performance: market structure, consolidation, and health care prices

**DOI:** 10.3389/fpubh.2023.1344939

**Published:** 2023-12-19

**Authors:** Glenn Melnick

**Affiliations:** University of Southern California, Los Angeles, CA, United States

**Keywords:** health care system, market structure, prices, transparency, policy options, health policies, concentration

This Research Topic includes research papers from two countries (United States and China) with different health care systems. Several universal themes emerge across the papers: the need for greater transparency and research to improve health system evaluation, the importance of a greater understanding of how health care prices affect consumer spending, and the role that government regulations might play in improving consumer welfare.

The article by Nie and Feng examines China's National Comprehensive Medical Reform (NCMR) pilot policy to study per capita medical expenses for inpatients and outpatients and the roles that hospital competition and institutional environment play in driving per capita medical expenses. The paper by Hermosilla et al. studies the link between COVID-19-induced unemployment and reductions in access to statin medications. Their study recognizes the potential role of price effects on consumers' ability to afford needed medications and the need for improved reporting and price transparency to fully explore these relationships. Gudiksen and Murray focus on the lack of price transparency in the US health care system—“prices vary in nearly incomprehensible ways that do not correlate with quality” and offer policy options to control rising health care prices in the United States. Montague et al. address the role of increased consolidation among healthcare providers, such as health systems, hospitals, and physicians on the healthcare market in the United States and the potential negative consequences of highly concentrated healthcare provider markets, including higher prices, mixed quality outcomes, and reduced access to healthcare services. The paper concludes with recommendations for broad policy interventions to address market failures and the importance of imposing conditions on healthcare provider transactions to restrain high and rising healthcare prices.

While there is substantial variation in health care systems across the globe including structure, financing, and operations, many of these systems share common trends and conditions. First, most of the world is facing an aging population contributing to higher chronic disease rates, greater need and utilization of health care services, and higher spending. These trends suggest an urgent and growing need for all countries to increase applied health policy research to manage a likely ever-increasing national health care budget.

The papers included in this Research Topic highlight the growing need for increased transparency and better reporting of data to support applied research to improve health care system effectiveness—across all countries.

## Author contributions

GM: Writing – original draft.

